# Failure Modes and Influencing Factors of Rubber O-Ring Seals in High-Pressure Hydrogen Environments

**DOI:** 10.3390/polym17223075

**Published:** 2025-11-20

**Authors:** Zhenwei Lv, Sohail Yasin, Jianfeng Shi, Sheng Zeng

**Affiliations:** 1 Hydrogen Energy Institute, Zhejiang University, Hangzhou 310027, China; 22460714@zju.edu.cn (Z.L.); shijianfeng@zju.edu.cn (J.S.); 2 Institute of Advanced Equipment, College of Energy Engineering, Zhejiang University, Hangzhou 310027, China; soh.yasin@gmail.com; 3 Engineering Research Center of High-Pressure Process Equipment and Safety, Ministry of Education, Zhejiang University, Hangzhou 310027, China

**Keywords:** O-rings, high-pressure hydrogen, failure modes, rubber, nanomaterials

## Abstract

Rubber O-rings play a crucial role in ensuring the safety and reliability of high-pressure hydrogen systems. However, their degradation and failure under hydrogen exposure remain a major barrier to the long-term stability of sealing structures. This review summarizes the failure modes of rubber O-rings in high-pressure hydrogen environments and clarifies the interaction mechanisms among hydrogen permeation, swelling, rapid gas decompression (RGD), and mechanical fatigue. Compared with conventional high-pressure gases, hydrogen significantly accelerates the coupling of mechanical and physicochemical degradation, leading to multi-mechanism failure characterized by blistering, crack propagation, and modulus reduction. This review highlights the limitations of existing research, including insufficient long-term experimental data, simplified single-mechanism models, and the lack of multi-physics coupling analysis. Future research priorities are proposed in four aspects: (1) development of hydrogen blister-resistant elastomers, (2) collaborative optimization of sealing structures and materials, (3) in-depth investigation of tribological behavior under hydrogen cycling, and (4) establishment of predictive life models integrating multi-scale simulations and experimental validation. This work provides a state of the art of hydrogen-induced failure mechanisms and offers theoretical and engineering guidance for designing reliable sealing systems in next-generation hydrogen energy applications.

## 1. Introduction

### 1.1. Application Background of O-Ring Seals in High-Pressure Hydrogen Environment

**Figure 1:**
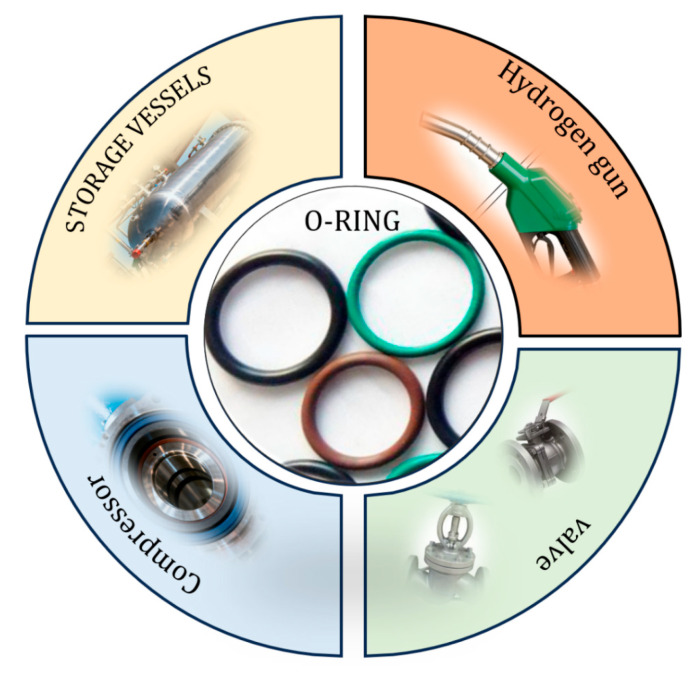
Common application scenarios of hydrogen environment O-ring seals.

With the gradual expansion of applications in the field of hydrogen energy [1,2,3], the storage and transportation of hydrogen impose stricter requirements on sealing systems [4]. In critical equipment such as hydrogen fuel cell vehicles, hydrogen refueling stations, and hydrogen storage tanks, O-ring seals have become the most commonly used and crucial sealing components due to their simple structure, low manufacturing cost, and ease of assembly [5,6,7]. Figure 1 depicts an illustration of O-rings used in various applications. The primary function of O-ring seals is to prevent hydrogen leakage and ensure the safe and stable operation of high-pressure hydrogen systems. However, the special physicochemical properties of hydrogen molecules can easily cause hydrogen-induced failure in O-rings, and this sealing weakness has become a key factor restricting the long-term stable operation of high-pressure hydrogen sealing systems.

In a high-pressure hydrogen environment, there are unique challenges to consider. The hydrogen molecule has a small molecular size and strong permeability, allowing it to easily enter and accumulate within rubber materials. Seals must operate under pressures of 70 MPa or higher while enduring frequent filling cycles, rapid depressurization processes, and temperature fluctuations. These factors combined can lead to material swelling, bubbling, cracking, and other failure modes [8]. Compared to a general high-pressure gas environment, failure mechanisms in a hydrogen environment are more complex and often involve multi-mechanism coupling. Seals must maintain stable sealing performance over extended periods under high-pressure hydrogen, while also coping with frequent filling cycles, rapid depressurization, and significant temperature variations. This requires seals to possess excellent sealing capability, adaptability, and resistance to chemical corrosion [9]. Therefore, exploring the design and material optimization of O-rings suitable for high-pressure hydrogen environments is of paramount importance for ensuring the stable and safe operation of hydrogen energy systems.

### 1.2. Objectives and Significance

A substantial body of research has been conducted on the influence of seal structure design and intrinsic material properties on failure mechanisms. But most of these studies focus on localized issues or short-term responses, lacking comprehensive analysis. Significant variations exist in the experimental conditions and evaluation criteria adopted by different studies, resulting in poor comparability between results and the absence of a unified conclusion. Furthermore, systematic experimental validation and theoretical analysis regarding failure mechanisms under the coupling effect of multiple mechanisms remain insufficient. These limitations lead to a fragmented and one-sided understanding, which is inadequate to provide comprehensive guidance for engineering applications.

In high-pressure hydrogen environments, O-ring seals often exhibit failure modes characterized by the superposition of multiple mechanisms, with distinct influencing factors underlying each mode. This article aims to systematically summarize the failure modes of O-rings in high-pressure hydrogen environments and their influencing factors, focusing on the classification of failure modes, influencing factors, performance evaluation criteria, and the interactions between various degradation mechanisms. The article highlights emerging hydrogen-specific failure modes, including hydrogen-induced swelling and RGD, as well as the exacerbation of traditional failure modes such as extrusion, wear, and compression deformation. In addition, the review summarizes the limitations of existing studies and outlines key research directions for future development to improve the reliability of hydrogen sealing systems.

## 2. Failure Modes and Evaluation Criteria

### 2.1. Common Failure Modes in High-Pressure Hydrogen Environment

**Figure 2:**
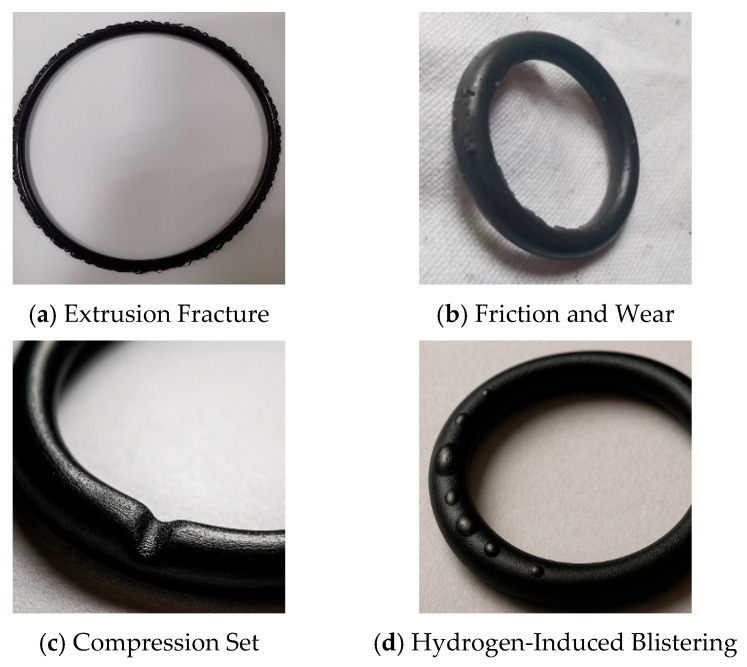
Common failure modes of O-rings in high-pressure hydrogen environments.

Under a high-pressure hydrogen environment, the failure behavior of O-rings often exhibits complex multi-mechanism characteristics, which can be mainly categorized into two types: mechanical damage caused by structural and load conditions, and material degradation induced by the hydrogen environment. Some of the hydrogen-induced O-ring failures are shown in Figure 2. Mechanical failure typically stems from structural design flaws, improper control of groove dimensions or compression ratio, with its typical modes including fatigue cracking, extrusion damage, wear, and stress cracking [9]. Fatigue damage is commonly observed in dynamic sealing systems; long-term repeated compression can induce microcracks in stress concentration areas, which gradually propagate and eventually lead to fracture or interface failure [10]. Extrusion damage, on the other hand, frequently occurs under high-pressure conditions when the groove design is unreasonable or the compression ratio is insufficient. In such cases, the rubber material is forced into the gap area, prone to tearing, shearing, or even complete ring detachment [11]. In working conditions where impurity particles, metal burrs, or eccentric movement of mating parts exist, long-term friction and fretting fatigue can cause surface wear and scratches, which further develop into crack sources and accelerate seal failure [11].

In addition to mechanical damage and failure, the physical and chemical effects inherent in the hydrogen environment pose more severe challenges to the stability of O-ring materials. The most catastrophic failure mode is rapid gas decompression (RGD) failure. Under a high-pressure hydrogen environment, hydrogen molecules permeate, dissolve, and diffuse within the rubber. When the system undergoes rapid decompression, these dissolved gases cannot escape in a timely manner, leading to the formation of high-pressure microcavities in the rubber microstructure. This, in turn, causes the material to blister, swell, or even burst [12,13]. Blistering and cracking reduce contact stress and the width of the sealing contact area, thereby significantly increasing the risk of leakage [14]. Such failures mostly occur under working conditions involving high-frequency opening/closing or periodic decompression, characterized by high suddenness and unpredictability. The permeation and diffusion of hydrogen are key factors contributing to hydrogen-induced blistering and fracture of rubber O-rings [15].

Hydrogen-induced swelling is also a crucial mechanism leading to failure. After hydrogen molecules enter the rubber, they cause volume expansion, which reduces the contact pressure and narrows the interface sealing width, ultimately resulting in seal leakage [15,16]. This process not only directly impairs sealing performance but may also exacerbate conventional failure modes such as extrusion and fatigue. Furthermore, under high-temperature and high-pressure conditions, rubber materials are prone to thermo-oxidative aging. The breakage of molecular chains and changes in cross-linked structures cause the material to harden and become brittle, which in turn leads to seal failure [17].

Overall, the failure of O-rings in a high-pressure hydrogen environment is characterized by the coexistence of newly emerging failure modes and an amplification effect. The newly emerging modes are mainly manifested as rapid decompression failure and hydrogen-induced swelling, while the amplification effect is reflected in the exacerbation of conventional modes (such as fatigue, extrusion, wear, and aging) under the influence of hydrogen. Among these, rapid decompression failure, due to its suddenness and thoroughness, is the most catastrophic dominant mode in a hydrogen environment and constitutes a critical bottleneck restricting the long-term reliable operation of sealing systems.

### 2.2. Failure Assessment Criteria

#### 2.2.1. Appearance and Geometric Dimensions

Changes in appearance and geometric dimensions are the most intuitive failure characteristics, suitable for early screening and pre-installation quality assessment. Cracks, fractures, and tears are the most common forms of appearance-related failure; therefore, focus should be placed on inspecting whether the surface of the seal ring and its installation area have traces such as scratches, impacts, or corrosion. Meanwhile, it is also necessary to measure whether the dimensions of the seal ring and related components meet the requirements. These failure phenomena are often associated with stress concentration, assembly interference, temperature shock, or material fatigue. Once obvious structural damage occurs, the seal ring can be directly determined as failed. The standard ISO 3601-3 lists several common defect types, such as flash, pits, inclusions, and scratches. This standard divides O-rings into different quality grades based on application requirements, most commonly Grade N (Normal) and Grade S (Special), which specify the maximum allowable defect size. For example, for O-rings with a cross-sectional diameter between 2.25 mm and 3.15 mm, the Grade N standard allows pits with a maximum diameter of no more than 0.08 mm. Additionally, performance failure can also be determined by Compression Set; when the compression set reaches 70%~80%, the seal ring can essentially be considered to have failed.

Apart from macroscopic observation, the vast majority of scholars currently prioritize a multi-scale approach, using microscopic detection techniques to track bubble changes [18,19,20], thereby revealing the essence of failure. These techniques can overcome the limitations of visual observation, accurately capturing the dynamics of bubbles within materials or at interfaces, providing direct evidence for failure attribution. By analyzing the morphology, composition, and distribution of bubbles, it is possible to distinguish whether the failure is caused by inherent material defects (such as residual bubbles from injection molding), operational conditions (such as gas release due to excessive temperature), or assembly issues (such as bubbles generated by extrusion), providing clear guidance for optimizing the design and use of sealing rings.

#### 2.2.2. Stress Analysis

When using finite element simulation to analyze the stress state of seal rings, the Von Mises equivalent stress is a critical indicator for evaluating whether elastomeric materials reach the yield state under multiaxial loading conditions. Although seal rings are generally made of rubber materials—whose constitutive behavior is typically hyperelastic—Von Mises stress is still widely used as an assessment criterion in many engineering assessments. It should be noted that rubber materials, strictly speaking, do not have a clear yield point. The Von Mises stress criterion is more of an engineering approximation method in rubber failure analysis, and its results should be evaluated in conjunction with experiments and other mechanical indicators to avoid one-sided conclusions. In particular, when the seal ring structure undergoes severe deformation, the assembly compression ratio is relatively high, or the sealing cavity has a complex geometry, local equivalent stress may concentrate and exceed the material’s tolerance limit, thereby causing tearing or shear yielding [21].

Maximum principal stress is commonly used to assess the fracture risk of sealing interfaces and is particularly suitable for analyzing tensile failure modes. When the sealing ring undergoes compressive deformation within the sealing cavity, it is first necessary to ensure that the contact stress meets the minimum sealing pressure required for high-pressure hydrogen environments. However, uneven distribution can lead to stress concentration at areas such as the lip edges and corners, resulting in maximum principal stress along the main axis. If this stress exceeds the tensile strength limit of the rubber, microcracks will propagate, eventually causing the sealing ring to fracture and fail. By combining the distribution maps of Von Mises stress, principal stress, and contact stress, a more comprehensive theoretical basis can be provided for designing the sealing cavity structure, selecting material hardness, and determining compression rate. Common engineering guidelines include the following: the contact stress must reach the minimum pressure required for sealing to prevent leakage; Von Mises stress should not exceed the yield strength of the material (e.g., about 6–10 MPa for nitrile rubber) to avoid permanent deformation and loss of sealing capability; the maximum principal stress must be kept below the tensile failure limit; and large stress gradients at edges or sealing lips should be avoided to prevent localized stress from exceeding the material’s performance limits.

#### 2.2.3. Leakage Rate Analysis

Leakage testing is the most direct assessment method, including air tightness tests and liquid tightness tests. Air tightness tests are usually conducted by observing pressure drop after pressurization or using gas leak detectors (e.g., helium mass spectrometry); liquid tightness tests involve applying pressure with hydraulic oil or water and checking for visible leakage. Table 1 summarizes the relevant standards, specifying in detail the leakage levels, test methods, and allowable values [22,23,24,25]. In common application scenarios—such as high-pressure hydrogen storage tanks for vehicles, hydrogen refueling station equipment, and hydrogen refueling nozzles—national standards have clearly defined performance requirements for O-rings in high-pressure hydrogen environments, including operating pressure, cycle count, sealing effect, and leakage rate. These standards are directly related to the safety, reliability, and long-term operational stability of hydrogen systems. The table below presents the key sealing performance requirements for O-rings in these application scenarios, providing important guidance for seal design and material selection.

### 2.3. Performance Evaluation Standards in High-Pressure Hydrogen Environments

Currently, the performance evaluation of elastomeric O-rings for high-pressure hydrogen environments follows a multi-tiered standard system. At the material level, standards quantify a rubber material’s resistance to hydrogen’s unique physical effects (such as high permeability and Rapid Gas Decompression, RGD). Industry-wide, NORSOK M-710 [26] and ISO 23936-2 [27] are widely adopted, assessing mechanical properties, volume changes post-hydrogen exposure, and specifically quantifying damage levels from RGD. Additionally, in automotive hydrogen storage applications, SAE J2579 [28] mandates stringent material compatibility tests, requiring materials to maintain critical mechanical properties and dimensional stability after exposure to 70 MPa hydrogen. For component performance standards, which validate macroscopic sealing performance under simulated operating conditions, this tier is enforced by top-level regulations such as GTR No. 13 (Global Technical Regulation) and reflected in specific component specifications, including standards for hydrogen refueling station valves (ISO 19880-3 [22]), hydrogen interfaces (SAE J2600 [29]), and onboard hydrogen storage vessels (GB/T 42612-2023 [25]). These standards explicitly define permissible leakage rate limits and durability lifespan requirements for sealing assemblies under extreme temperatures and high-frequency pressure cycling conditions. The synergistic effect of these standards aims to ensure the full life-cycle safety of O-rings in harsh hydrogen environments, from the micro-scale compatibility of materials to the macro-scale reliability of components.

## 3. Main Failure Modes Under Conventional High-Pressure Environment

### 3.1. Extrusion and Nibbling

Extrusion and nibbling are among the most typical and destructive failure modes of O-rings in a conventional high-pressure environment. Their main characteristic is that the rubber material is forced into the groove gap under high pressure, resulting in local tearing, shearing, or even complete ring detachment, often accompanied by obvious bite marks or notches on the surface. Once such failures occur, they usually lead to irreversible seal failure and may induce serious leakage accidents in a short time. Existing studies consistently suggest that extrusion is a flexural failure process caused by insufficient edge constraint, and it is one of the most probable failure modes for seals [30]. Its formation mechanism mainly stems from the imbalance between edge forces and constraints. When the groove design is unreasonable or the lip compression is insufficient, stress concentration tends to occur in the edge area, causing cracks to initiate and propagate rapidly under high pressure. Under such conditions, even if the overall compression rate is within the recommended range, local areas may still experience material being forcibly pushed into gaps, eventually forming nibbling or tearing.

Experimental and simulation results by Zhu et al. [31] indicate that extrusion often initially appears as slight tearing of the lip, gradually developing into macroscopic nibbling under pressure relief or cyclic loading, and ultimately leading to complete loss of sealing function. Although extrusion sensitivity is influenced by factors such as groove gap, compression ratio, and material hardness, these factors mostly regulate the conditions for failure occurrence rather than changing its basic mechanism. Therefore, engineering efforts to control extrusion failure should focus on optimizing local structural design and stress distribution, such as appropriately selecting compression ratios, improving groove geometric transitions, and adopting anti-extrusion structures like backup rings [32,33,34,35,36], rather than simply relying on increasing material hardness to delay failure.

### 3.2. Fretting Wear and Crack Evolution

In a conventional high-pressure environment, fretting wear and frictional fatigue are among the typical and non-negligible failure modes of O-rings. Their main characteristic is that when there is a slight relative displacement or vibration between the seal and the mating part, the contact interface is gradually damaged under repeated shear action, eventually forming crack sources which then evolve into leakage channels. The main inducements of fretting wear include excessively high surface roughness of the mating part, assembly eccentricity or insufficient coaxiality, and the presence of particulate matter in the medium. When these factors overlap, the rubber surface is more prone to scratches, cuts, or local pitting, which then become crack initiation sites and accelerate the propagation of fatigue cracks [37,38]. Studies by Zhou et al. have shown that after particle intrusion, the wear morphology of rubber seals gradually changes from mild abrasive wear to severe spalling, and surface cracks rapidly propagate into leakage channels [37]; further research has indicated that the hard/soft interface undergoes early failure under the action of particles, significantly shortening the service life of O-rings [38].

Existing studies generally suggest that the evolution of fretting fatigue typically goes through three stages: initiation—steady state—acceleration. In the initiation stage, interface damage is mainly characterized by slight scratches; in the steady state stage, fatigue cracks gradually propagate stably; in the acceleration stage, cracks interconnect and form channels, leading to a significant decline in sealing performance [37]. Finite element and experimental analyses have shown that under conditions such as excessively high compression ratio, unreasonable geometric design, or low temperature, interface stress concentration occurs earlier, and the time for cracks to enter the acceleration stage is significantly shortened. Through modeling research, Song pointed out that the contact stress distribution of large-sized O-rings under complex loads is extremely uneven, which is the main driving force for the early propagation of fatigue cracks [39].

In addition, working conditions and material formulations have a significant regulatory effect on fretting wear and crack evolution. Experimental results by Liu et al. have shown that silicone rubber undergoes severe spalling under dry friction conditions, while crack initiation is significantly delayed under lubricated conditions [40]. Kuang et al. found that the combination of fillers and plasticizers can stabilize the friction coefficient and reduce the wear rate, thereby delaying crack propagation [41]. Qiao et al. conducted a comparative study and found that different types of rubbers (such as nitrile butadiene rubber (NBR) and fluoroelastomer (FKM)) have significant differences in wear rate under reciprocating friction, and FKM exhibits stronger wear resistance and crack resistance under high-temperature conditions [42]. These results indicate that fretting wear and crack evolution are not a single process, but the result of the combined action of multiple factors such as structural stress, particle action, interface state, and material formulation.

**Figure 3:**
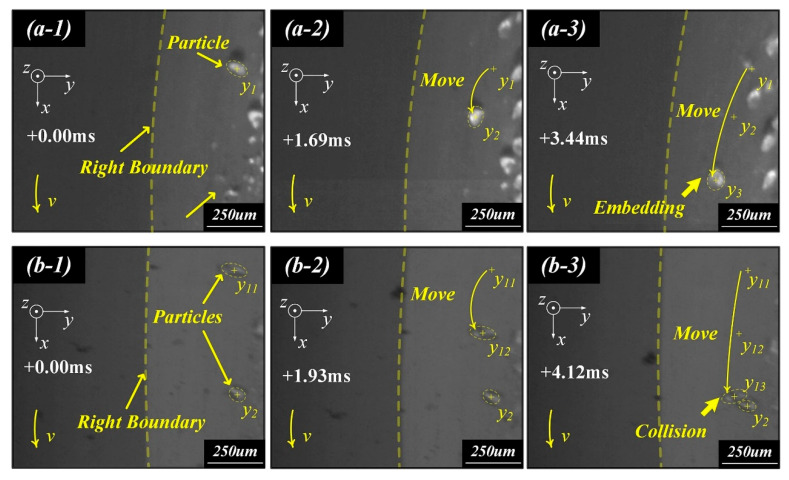
Motion of particles. (**a**-**1**) Particle contact with the rubber surface at 0.00 ms. (**a**-**2**) Particle moved at 1.69 ms. (**a**-**3**) Particle embedded at 3.44 ms. (**b**-**1**) Two particles at 0.00 ms. (**b**-**2**) Two particles were close together at 1.93 ms. (**b**-**3**) Particle collision at 4.12 ms [38].

As illustrated in Figure 3, the dynamic evolution process of particle–rubber interactions can be divided into several distinct stages: initial surface contact, gradual embedding into the elastomer matrix, and subsequent inter-particle collision or coalescence. During these interaction processes, localized stress concentration and micro-cutting effects emerge on the rubber surface, leading to the initiation of microcracks, which progressively propagate into deeper regions. This accumulated fatigue ultimately forms an interconnected crack network and leakage channels, thereby accelerating the degradation of sealing performance under cyclic loading conditions [38].

This observation confirms that fretting fatigue is a cumulative process driven by both mechanical and interfacial factors. Not only does it explain the typical wear morphologies observed in experiments, but it also establishes a mechanistic bridge for subsequent analyses of material formulations and anti-wear design strategies.

To address this failure mode, a variety of measures are commonly used in engineering practice to delay or suppress its occurrence: improving the machining quality of mating surfaces to avoid burrs and excessively high roughness; ensuring assembly coaxiality to reduce uneven loads; controlling the compression ratio within a reasonable range to avoid inducing fatigue cracks due to insufficient resilience or stress concentration; and selecting material formulations with higher cross-linking density and better wear resistance to fundamentally enhance crack resistance.

### 3.3. Thermal Aging and Compression Set

Thermal aging mainly refers to the phenomenon where the molecular chains of rubber materials break or the cross-linking density changes under the combined action of temperature and oxygen, thereby leading to a decline in mechanical properties. Studies by Mostafa et al. have shown that oil and thermal aging have a significant impact on the sealing performance of NBR O-rings; in particular, as temperature increases, changes in cross-linking density significantly affect their mechanical and durability properties [43]. Oxidative aging in high-temperature environments can significantly reduce the service life of O-ring seals [44]. In the initial stage, the tensile strength and resilience of the material gradually decrease; as aging time extends, the rubber gradually hardens and becomes brittle, and eventually surface cracking and fragmentation occur, which directly impairs its sealing function [17]. Through accelerated aging tests and service life prediction, Pan found that the elasticity and elongation at break of NBR O-rings decreased significantly after high-temperature aging, which poses challenges to their long-term storage and service life [45].

A compression set refers to the loss of resilience of O-rings under long-term compressive stress and temperature. Its essence is the irreversible relaxation of material molecular chains under the coupling effect of heat and force, making it impossible to restore the original shape after unloading. This property is usually tested in accordance with GB/T 7759 or ASTM D395 [46,47] standards to evaluate the resilience of rubber materials during service. For O-rings, their sealing effect relies on the contact stress generated by pre-compression in the groove to prevent gas leakage. Once the O-ring cannot fully rebound after compression, separation of the contact surface or insufficient pre-tightening force will occur in the sealing groove, thereby forming a leakage channel. Conversely, the design of compression ratio and structural parameters also has a coupled impact on the compression set behavior. An increase in compression set will significantly reduce the sealing contact stress, making the interface more prone to leakage [7,48].

In practical operation, thermal aging and compression set are often coupled with each other. Thermal aging reduces the recovery capacity of molecular chains, thereby accelerating the accumulation of compression set, whereas the development of compression set weakens the interface contact pressure, making the material deteriorate faster under thermal stress. In engineering applications, measures to delay thermal aging and compression set mainly include the following: controlling the service temperature to avoid long-term exposure to environment above the heat-resistant limit of the material; improving the thermal-oxidative aging resistance of rubber through formula modification, such as adding antioxidants, stabilizers, and heat-resistant fillers; optimizing the compression ratio design to avoid rapid accumulation of compression set caused by excessively high or low ratios; and conducting regular inspection, maintenance, and replacement of seals to prevent failure due to excessive compression set.

In conventional high-pressure environments, the failure modes of O-rings mainly include extrusion and nibbling, fretting wear and crack evolution, as well as thermal aging and compression set. These failure modes rarely exist independently in actual working conditions; instead, they are interrelated. For instance, thermal aging reduces the elasticity of rubber and accelerates compression set; surface wear and scratches provide potential defect sources for crack initiation; and an increase in compression set lowers the contact pressure ratio, making the interface more prone to entering the stages of frictional fatigue or crack propagation. Failure in conventional environments is not an isolated event but the result of the superposition of multiple mechanisms. On this basis, when the sealing environment changes from ordinary high-pressure gas to high-pressure hydrogen, the existing failure modes are not only amplified, but new damage mechanisms also emerge, making the failure modes more complex.

## 4. Failure Modes in High-Pressure Hydrogen Environment

### 4.1. Overview

When the sealing environment changes from ordinary high-pressure gas to high-pressure hydrogen, the failure mechanism of O-rings exhibits higher complexity and specificity. During service, hydrogen continuously penetrates the rubber matrix and gradually accumulates within it. At the same time, frequent charging and depressurization cycles intensify the desorption and diffusion processes of hydrogen. Under such effects, O-rings not only experience failure modes existing in conventional high-pressure environments (such as extrusion, wear, thermal aging, and compression set), but also show a significant increase in the evolution rate and damage degree of these modes. Meanwhile, the high-pressure hydrogen environment can also induce new failure modes that are hardly observable under conventional conditions, such as blistering and cracking caused by rapid depressurization, and hydrogen-induced swelling and permeation. These failure forms are characterized by strong suddenness, difficulty in prediction, and obvious cumulative effects, which seriously threaten the long-term reliability of the sealing system. Therefore, a comprehensive analysis of the new failure modes and the amplification effect of conventional modes in a hydrogen environment is of great significance for revealing the failure law of O-rings in hydrogen energy equipment.

### 4.2. Performance of Typical Rubber Materials in High-Pressure Hydrogen Environments

In high-pressure hydrogen facilities, the material of the sealing ring is crucial. Different materials have varying permeability and diffusion coefficients, resulting in significant differences in the severity of hydrogen molecule damage. Currently, there are three main types of sealing rings used in high-pressure hydrogen environments: NBR, ethylene propylene diene monomer rubber (EPDM), and FKM.

Some scholars [8] systematically evaluated the physical and mechanical properties of three typical rubber materials—NBR, EPDM, and FKM—in high-pressure hydrogen environments at 35 MPa and 70 MPa. The results showed that EPDM had the highest hydrogen permeability coefficient (17.0 × 10^−9^ mol/m·s·MPa) and diffusion coefficient (5.2 × 10^−10^ m^2^/s), while FKM had the lowest values for both (2.1 × 10^−9^ mol/m·s·MPa and 0.8 × 10^−10^ m^2^/s, respectively), demonstrating excellent gas tightness. In terms of volume swelling and hydrogen content, NBR absorbed the most hydrogen (approximately 950 ppm) but exhibited the smallest volumetric expansion (relative change below 10%), whereas FKM, although absorbing the least hydrogen (approximately 430 ppm), experienced a volume expansion exceeding 60% under 70 MPa with a recovery time as long as 83 h; EPDM showed the fastest volume recovery, requiring only 4 h, while FKM needed 51 h. Regarding mechanical properties, all materials showed a significant decrease in tensile strength after exposure, with NBR and FKM lagging behind in recovery relative to volume change, indicating that retained internal hydrogen and structural damage affected their mechanical performance recovery. Although changes in compression set (CS) were not significant, SEM images revealed that both NBR and FKM developed cracks under 70 MPa. Additionally, the study found a power–law relationship between relative true stress and volume ratio (σ_T_/σ_To_ = (V_o_/V)^α), with α values of NBR: 1.53, EPDM: 1.05, and FKM: 1.23, revealing that volume change is a key driving factor for mechanical performance degradation rather than surface crack formation. These findings provide important guidance for the material selection and lifespan assessment of O-rings in high-pressure hydrogen applications.

### 4.3. Newly Emerged Failure Modes in Hydrogen Environment

#### 4.3.1. Hydrogen-Induced Swelling

According to Fick’s Law, in high-pressure hydrogen environments, the gradient of hydrogen concentration drives the diffusion of hydrogen molecules in rubber and triggers volume expansion [49]. This expansion process disrupts the initial contact stress distribution at the sealing interface, causing the stress to shift from a U-shape to a V-shape. As a result, the effective preload in the central area of the sealing surface is reduced, significantly increasing the risk of leakage [35].

**Figure 4:**
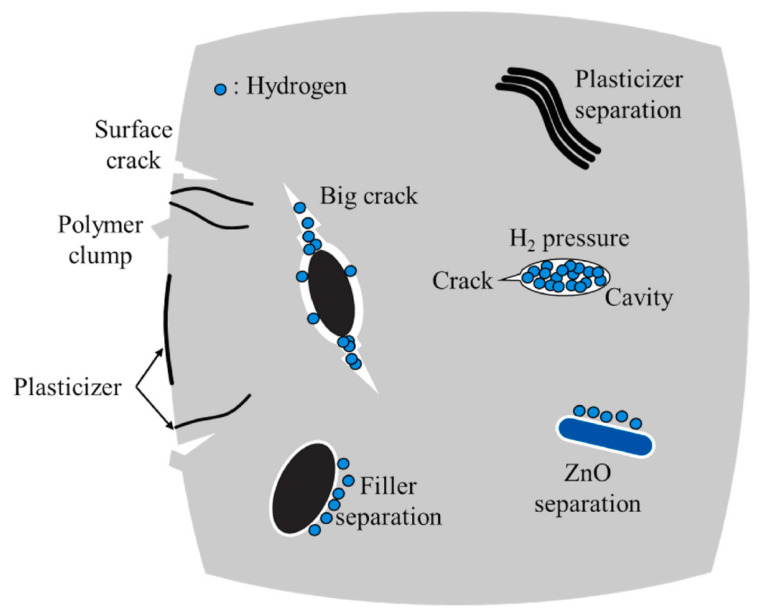
Illustration of micromorphology damage of rubber under hydrogen-induced swelling [16].

As shown in Figure 4, the microstructure of rubber undergoes significant changes under hydrogen-induced swelling. The dissolution and diffusion of hydrogen molecules within the material lead to the formation of microvoids and cracks, manifested as interfacial delamination and volumetric expansion. Such microscopic damage weakens the elastic modulus and recovery ability of the rubber, thereby causing a reduction in contact stress and eventual sealing failure at the macroscopic level.

**Figure 5:**
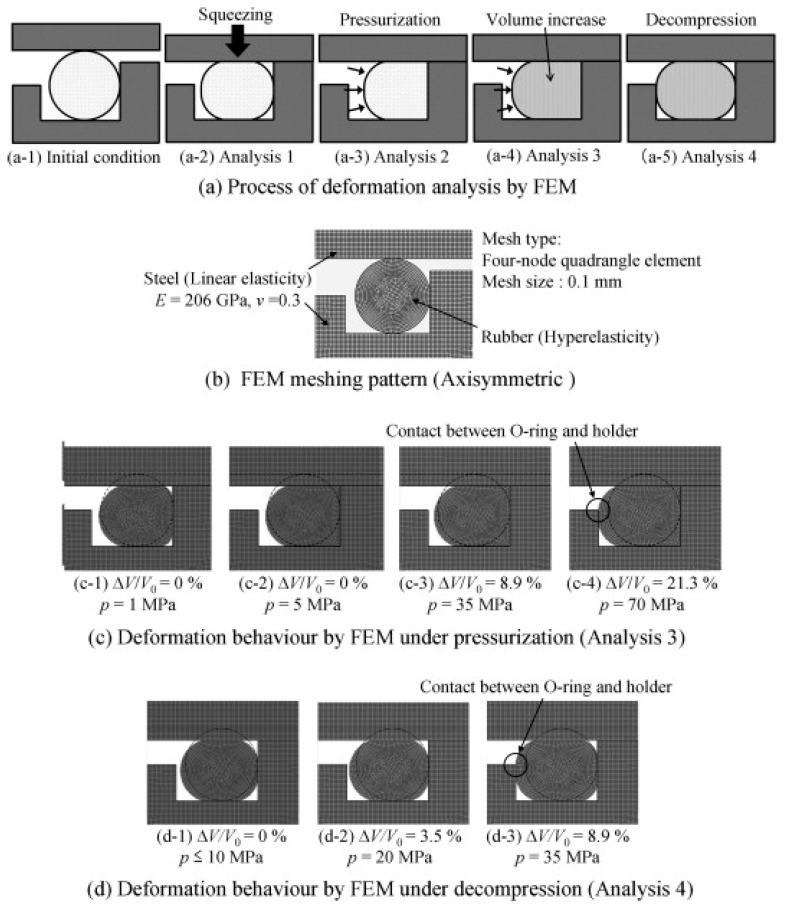
Time-dependent volume changes in O-rings after hydrogen exposure [52]. (**a**) Schematic diagram of the deformation analysis process using finite element calculation. (**b**) Mesh generation of the finite element model. (**c**) Deformed state during the high-pressure pressurization phase (Analysis 3). (**d**) Deformed state during the depressurization phase (Analysis 4).

After dissolution, hydrogen causes swelling inside the rubber. With the accumulation of hydrogen, the material’s elastic modulus and resilience decrease, which gradually diminishes the original sealing effect of the sealing surface during long-term service. Hydrogen-induced swelling and permeation failure are progressive processes, and it usually takes a certain amount of time for their weakening effect on material performance to become apparent. Studies have shown that hydrogen solubility and diffusion coefficient have a significant impact on swelling behavior: the higher the hydrogen solubility and the lower the diffusion coefficient, the more pronounced the accumulation of hydrogen inside the rubber. This leads to a significant enhancement of the swelling effect, thereby accelerating material failure [50], and the hydrogen permeation process is mainly influenced by the diffusion coefficient [51]. Figure 5 shows the volume changes in the O-ring over time after exposure to hydrogen.

In addition to these effects, the deformation characteristics of O-rings in hydrogen environments deserve further attention. The ingress and accumulation of hydrogen molecules within the elastomer alter its elastic modulus and bulk compressibility, resulting in a redistribution of contact pressure at the sealing interface. Finite element studies have revealed that hydrogen-induced swelling and modulus reduction lead to non-uniform deformation, where local strain concentrations emerge near groove corners and lip edges. Such localized deformation accelerates crack initiation and propagation under cyclic loading [7,53]. Moreover, the coupling of mechanical compression and hydrogen diffusion causes time-dependent relaxation behavior, contributing to permanent deformation (compression set) and gradual loss of sealing contact pressure during long-term service. Therefore, analyzing the deformation response of O-rings under hydrogen exposure is crucial for understanding and predicting their failure evolution.

The swelling behavior of materials is closely related to factors such as hydrogen solubility, diffusion coefficient, filler type, cavity size and distribution, operating pressure, and temperature. Numerical simulation studies have revealed that the diffusion behavior of hydrogen inside the material interacts with microcavity defects in the material, resulting in strain concentration and potentially triggering crack propagation—further exacerbating the occurrence of failure [49]. Therefore, material selection is crucial: priority should be given to sealing materials with high diffusion coefficients and low solubility to slow down the accumulation of hydrogen in rubber and the swelling effect. To ensure the reliability of sealing structures in high-pressure hydrogen environments, it is also necessary to optimize the compression ratio and preload of O-rings and strictly control the depressurization rate to mitigate the negative impacts of hydrogen swelling and permeation processes [50].

Material compatibility plays a crucial role in ensuring sealing reliability in high-pressure hydrogen environments. Studies have shown that traditional elastomers, NBR, are prone to excessive hydrogen permeation and swelling, leading to blistering and rapid degradation of mechanical integrity. In contrast, FKM and polytetrafluoroethylene (PTFE) exhibit superior hydrogen resistance due to their dense molecular chains and low free volume, which effectively suppresses hydrogen diffusion and chemical interaction with the polymer matrix [54,55]. Therefore, these materials are recommended for hydrogen service applications requiring long-term stability and low leakage.

#### 4.3.2. Rapid Gas Decompression

**Figure 6:**
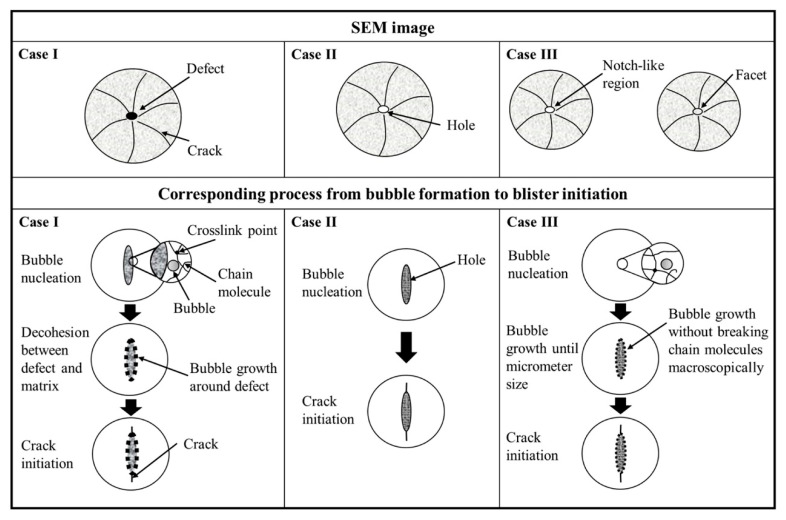
Schematic diagram of the process from bubble formation to blister initiation [60].

**Figure 7:**
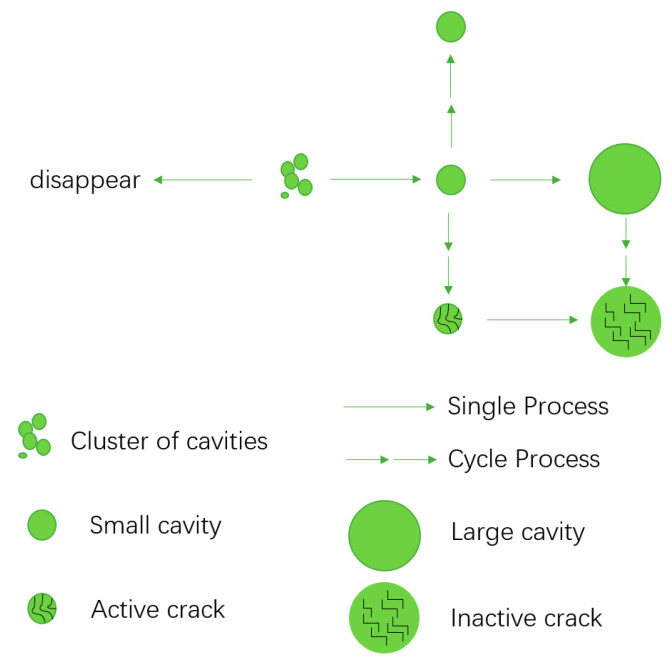
Evolution model of blister during cyclic hydrogen exposure [61].

Rapid Gas Decompression (RGD) failure is one of the most typical and catastrophic failure modes of O-rings in high-pressure hydrogen environments. When rubber materials are exposed to high-pressure hydrogen for a long time, a large number of hydrogen molecules can permeate and dissolve into the matrix. During the rapid depressurization process, these molecules have no time to diffuse and escape, thus forming high-pressure gas cavities in the material microstructure, which trigger blistering, swelling, and even crack penetration [56,57]. This process usually follows the evolution path of permeation—dissolution—accumulation—release, and its severity is closely related to the rubber’s own molecular structure, filler properties, and service conditions [58,59]. For example, NBR exhibits a relatively high permeation risk in hydrogen, while hydrogenated nitrile rubber (HNBR) and FKM have denser molecular chains and smaller free volumes, resulting in stronger resistance to permeation and blistering [56]. Filler type also has a significant impact: silica enhances hydrogen solubility and increases the risk of blistering, while carbon black systems relatively inhibit this process [59]. Figure 6 and Figure 7 illustrate the entire process of bubble formation and blister development due to hydrogen permeation. As hydrogen accumulates within the rubber material, microvoids gradually expand, leading to blister formation. Under cyclic hydrogen exposure, blister growth accelerates, eventually resulting in a network of cracks that significantly increases the risk of sealing failure.

Kuang [62] conducted an analysis from a microscopic perspective and found that the microscopic damage of EPDM O-rings under high-pressure hydrogen begins at the interface between ZnO agglomerates and the matrix (an inherent weak point): hydrogen causes partial conversion of ZnO to ZnS, sulfur elements migrate and form, weakening the interfacial bonding and leading to the initiation of 100–350 nm nanopores. Pressure cycling connects the pores into micron-sized dendritic cracks, migration of the plasticizer DOS induces surface microcracks, and during RGD, hydrogen molecule expansion causes sudden crack propagation. Fragmented ZnO particles exacerbate wear, ultimately resulting in microstructural collapse that triggers macroscopic seal failure.

External operating conditions often directly determine the trigger threshold and evolution rate of RGD failure. Excessively fast depressurization rate leaves dissolved hydrogen no time to escape, leading to rapid accumulation of bubbles and strong stress concentration in defect areas, which accelerates blister rupture and crack propagation [34,59,63]. Temperature has a dual effect: increasing temperature helps accelerate the diffusion and escape of hydrogen, but at the same time weakens the mechanical strength of the matrix, which may instead increase the failure risk under certain conditions [58]. In practical applications, O-rings usually face high-frequency hydrogen charging and discharging cycles, and the cumulative damage effect is far more severe than that of a single depressurization. Each hydrogen cycle leads to hydrogen residue and microcavity expansion, with the number and volume of blisters increasing accordingly. Eventually, a crack network is formed and permeation channels are reconstructed, showing significant nonlinear cumulative characteristics, thus greatly shortening the service life [64].

To evaluate the resistance of sealing materials to RGD failure, international standardized testing methods such as NORSOK M-710 and ISO 23936-2 [26,27] are commonly used. These methods typically involve applying high-pressure gas to the sample, setting the depressurization rate, and then grading the sample based on the internal crack level. For the observation of cracks, the vast majority of scholars currently use SEM for observation [65,66].

For the prevention and control of RGD, the main approaches currently include material and structure optimization, as well as operating condition control. At the material level, increasing cross-linking density, using multi-component blending, or introducing nano-fillers can effectively reduce hydrogen solubility or increase diffusion rate [67,68,69,70], thereby alleviating hydrogen accumulation in the matrix. Nano-fillers, being an integral part of rubbers [71,72,73,74,75], especially in terms of structure and process [76,77,78,79,80,81], should be used to reduce initial internal defects and residual stress in the material, thereby lowering the probability of gas cavity initiation. Meanwhile, reasonably setting the depressurization rate and pressure cycle amplitude are also key operational measures. In the future, it is necessary to establish unified blister evaluation indicators and service life prediction models to achieve more reliable seal design and material selection.

### 4.4. Amplification Effect of Conventional Failure Modes in Hydrogen Environment

**Figure 8:**
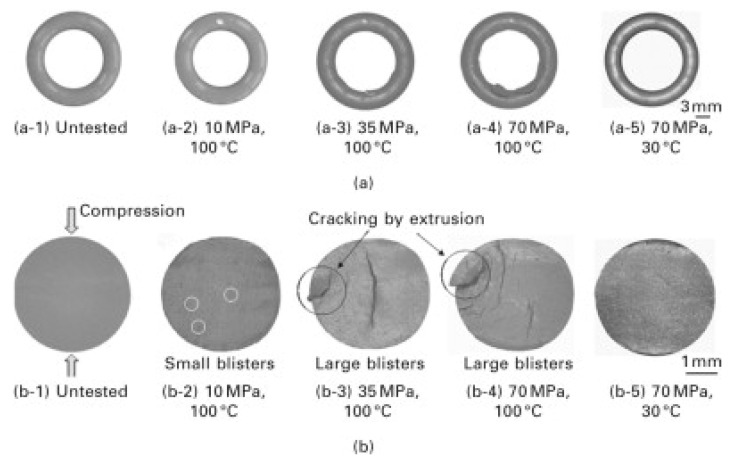
Examples of crack initiation conditions of O-ring specimens: (**a**) appearance; (**b**) cross-section [15].

Under high-pressure hydrogen environments, common failure modes of O-rings generally exhibit more pronounced amplification effects, and their evolution process is also more complex. Hydrogen permeation reduces the modulus and hardness of rubber, causing a significant decrease in the critical extrusion pressure and making edge cracks more likely to propagate into macroscopic biting or tearing. This implies that in a hydrogen environment, even relatively well-designed structures are more prone to losing constraint stability under high pressure. This vulnerability is reflected not only at structural edges but also in the friction behavior at interfaces. Zhou et al. [14] established a dynamic finite element model based on the Mooney–Rivlin constitutive model and the Fick diffusion model to simulate the fretting fatigue behavior of NBR O-rings under hydrogen exposure. The results indicate that hydrogen permeation causes the rubber to expand in volume, leading to a redistribution of the contact stress at the sealing lip. Due to hydrogen permeation, the O-ring surface is more likely to enter the “sticking regime”, where frictional energy is concentrated and released in narrow regions, increasing the risk of crack propagation. Furthermore, the impact of hydrogen permeation on the internal Mises stress of the O-ring is significant. In particular, in high-pressure hydrogen environments with hydrogen pressures of 35 MPa, the peak Mises stress fluctuates periodically, accelerating crack propagation and degrading the O-ring’s sealing performance. Figure 8 shows the performance of rubber O-ring samples under different pressure and temperature conditions, illustrating their deformation (compression and bulging) and cracking behavior under various experimental conditions.

In a hydrogen environment, the effect of thermal aging on sealing materials, particularly O-rings, is significant. Hydrogen permeation exacerbates the thermal aging effects of rubber materials, manifested by a reduction in hardness and modulus, which in turn affects their sealing performance. As the temperature increases, hydrogen accelerates the thermal aging process of rubber materials [82]. Under high-temperature conditions, the compression set of rubber materials increases, especially when temperatures exceed a certain threshold, where the thermal aging effects become more pronounced. Due to the presence of hydrogen, the material’s elasticity and recovery capacity are diminished, leading to incomplete rebound of the O-ring, thereby compromising its sealing performance. Temperature fluctuations during the thermal aging process can induce structural changes within the material, which further accelerates hydrogen permeation and the degradation of rubber properties. Overall, the combined effect of high temperature and hydrogen significantly accelerates the aging of O-rings in hydrogen environments, affecting their sealing performance over long-term use.

### 4.5. Multi-Factor Coupling Effects in High-Pressure Hydrogen Environments

Under high-pressure hydrogen environments, the actual working conditions are not influenced by a single factor but by the coupling and superposition of multiple factors. Currently, the vast majority of scholars conduct research using simulation methods.

**Figure 9:**
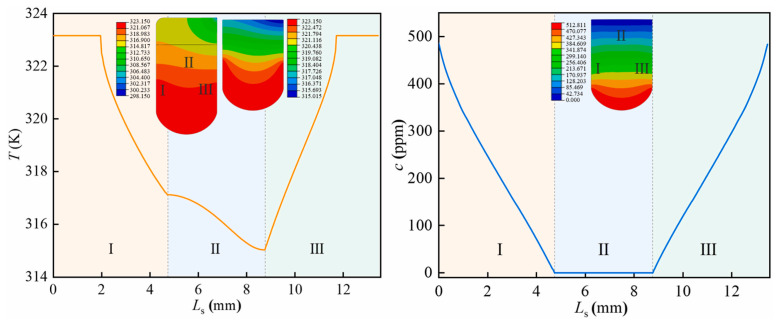
Internal temperature and hydrogen concentration under thermo-mechanical-diffusion coupling [83].

Ma [83] focused on the coupling of thermal, mechanical, and hydrogen diffusion fields in nitrile rubber (NBR), clarifying the interaction rules among the three: temperature regulates mechanical properties and hydrogen diffusion rate by altering the activity of rubber molecular chains, mechanical stress guides directional migration of hydrogen molecules, and the concentration gradient formed by hydrogen diffusion provides feedback on stress transmission. The study found that under the coupling of these three fields, the maximum contact stress and Von Mises stress of O-rings increased by less than 5% compared to purely high-pressure mechanical fields. Moreover, when hydrogen pressure rises to 70 MPa, sudden changes in the concentration gradient caused by hydrogen diffusion significantly increase the probability of rubber failure, and the driving force of the concentration gradient on hydrogen diffusion outweighs the effects of temperature promotion and stress inhibition. Figure 9 shows the distribution of temperature and hydrogen concentration (T, c) inside the rubber seal under thermo-mechanical-diffusion coupling, and presents the trend of these parameters along the low-pressure side (L_s_) sealing surface. Ma [84] extended the basic coupling to transient conditions, analyzing the dynamic coupling of transient hydrogen pressure, hydrogen charging-induced heat generation, and mechanical deformation: increasing hydrogen charging rate (0.01~0.04 kg/s) accelerates hydrogen penetration and heat accumulation, causing the NBR O-ring contact width to increase from 9 mm to 14 mm (a 55% increase) and the maximum contact pressure to rise from 10.3 MPa to 77.1 MPa (a 6.5-fold increase). Qiao [35] further deepened the study of dynamic coupling, focusing on the effect of the interaction between hydrogen permeation and mechanical stress on distribution: when hydrogen pressure increases from 10 MPa to 70 MPa, hydrogen penetration alters the stress transmission path inside NBR, with the kurtosis coefficient increasing from 1.2 to 1.7, intensifying stress concentration. Conversely, when the compression ratio increases within the range of 8–20%, the constraint of the mechanical field on hydrogen permeation is enhanced, which can improve stress distribution uniformity and reduce local failure risk. Zhang [85] focused on FKM sealing structure of 70 MPa Type IV hydrogen storage cylinders, mainly investigating the combined effects of three factors: thermal field (temperature: 25~100 °C), mechanical field (hydrogen pressure: 10~70 MPa and sealing interface stress/contact pressure), and hydrogen-induced expansion, as well as the regulation of seal groove structural parameters on this coupling effect. It is found that an increase in temperature reduces the elastic modulus of FKM, resulting in a non-linear decrease in the seal’s maximum stress (with a maximum reduction of 40%), contact pressure (with a maximum reduction of 20%), and shear stress (with a maximum reduction of 24%). Furthermore, the higher the hydrogen pressure (e.g., 70 MPa), the smaller the reduction in contact pressure caused by temperature (only 5.18%), which is due to high pressure inhibiting the thermal expansion of rubber. In contrast, hydrogen-induced expansion increases contact pressure while intensifying stress concentration. Chen [57] simulated the process of hydrogen diffusion and the deformation of cavity defects within rubber O-rings, revealing the coupling effects between hydrogen diffusion, internal cavity pressure, hydrogen-induced swelling, and rubber deformation. Hydrogen diffusion leads to an increase in cavity pressure, which in turn causes changes in cavity size and material deformation, with hydrogen-induced swelling further exacerbating this process. Through a comparative analysis of different rubber materials (such as EPDM, NBR, FKM, etc.), it was found that materials with higher hydrogen diffusion coefficients can reach a steady state more quickly, reducing cavity expansion and strain accumulation, thereby lowering the risk of material rupture.

Among all the coupling relationships mentioned above, hydrogen permeation is always the core link that connects various physical fields in series and affects sealing performance. Hydrogen permeation, driven by concentration gradients, dominates the direction of diffusion and determines the intensity of multi-field interactions. Under transient hydrogen charging conditions, the hydrogen permeation rate dynamically changes with charging parameters, directly causing coupled fluctuations in pressure, temperature, and deformation. In studies of dynamic stress distribution, hydrogen permeation alters stress transfer paths and is the fundamental cause of intensified stress concentration. In engineering scenarios, hydrogen permeation-induced swelling is a key factor in FKM performance degradation and pressure fluctuations. The failure risk of defect-containing seals also stems from the enrichment effect of hydrogen permeation in defect regions. Therefore, hydrogen permeation is not only the core variable in the multi-field coupling of rubber O-rings in high-pressure hydrogen environments but also a critical regulatory factor that determines sealing reliability, service life, and failure probability, and it must be considered as a central concern in theoretical analysis and engineering design.

## 5. Future Research Directions and Challenges

### 5.1. Existing Problems in Current Research

With the rapid advancement of hydrogen energy technology, sealing technology in high-pressure hydrogen environments—especially research on rubber O-ring seals—has become a key link in ensuring the safe and reliable operation of hydrogen energy systems. Although certain research has been conducted on the failure modes of rubber O-rings in high-pressure hydrogen environments, obvious gaps still exist. Most studies focus on the discussion of single failure modes, such as hydrogen-induced swelling, blistering and fracture, and thermal aging. However, these failure modes usually do not exist independently; instead, they result from the coupling of multiple mechanisms. Therefore, current research lacks an in-depth exploration of the interactions between different failure mechanisms, particularly the failure process under the interweaving of multiple factors, such as hydrogen permeation, stress concentration, and friction and wear.

Existing experimental studies are mostly concentrated on short-term tests under single working conditions, and there is a lack of investigation into the failure evolution process of sealing systems during long-term operation in high-pressure hydrogen environments. In particular, systematic data on changes in material performance under complex working conditions—such as high pressure, frequent charging and depressurization, and dynamic loads—is insufficient. Furthermore, although numerical simulation is increasingly applied in a hydrogen environment, most studies remain at the stage of single-physics-field simulation and fail to effectively couple and conduct in-depth analysis of multiple physical processes, such as hydrogen permeation, stress fields, and fretting fatigue. This limits the accuracy and practicality of existing failure prediction models in engineering applications. Research on material optimization and working condition matching also has shortcomings. Current studies have not fully considered the accumulation effect of hydrogen during multiple hydrogen charging–discharging cycles and rapid depressurization processes. Additionally, there is insufficient research on the impact of key material parameters—such as surface modification, filler selection, and cross-linking degree adjustment—on sealing performance in a hydrogen environment. To summarize, while existing research has provided a preliminary understanding of rubber O-rings in high-pressure hydrogen environments, issues such as insufficient exploration of multi-mechanism coupling, lack of long-term dynamic experimental data, and inadequate model accuracy still exist, which need to be further addressed in future research.

### 5.2. Key Future Research Directions

#### 5.2.1. R&D of High-Performance Blister-Resistant Rubber Materials

Under working conditions involving multiple hydrogen cycles or fluctuations in high pressure and high temperature, rubber materials are highly prone to blistering failure, which has become one of the major technical bottlenecks limiting current sealing reliability. Future efforts should focus on the research and development of hydrogen blister-resistant rubber materials, particularly FKM- and PTFE-based composites with enhanced cross-linking density and optimized filler systems to reduce hydrogen solubility and improve resistance to rapid gas decompression and enhance their capabilities of resisting hydrogen permeation, hydrogen release, and blister growth through approaches such as polymer modification and filler optimization.

#### 5.2.2. Collaborative Design Optimization of Sealing Structures

O-rings in high-pressure hydrogen environments are simultaneously subjected to multiple factors such as mechanical loads, hydrogen permeation, and temperature changes. Optimization of a single parameter can hardly meet engineering requirements anymore. Future research should focus on the combined effects of different overlapping factors on sealing performance and service life. A multi-factor coupling model can be established, considering wear, material properties, temperature fluctuations, and cyclic loads, combined with actual operating conditions to achieve an accurate prediction of seal performance degradation trends and service life.

#### 5.2.3. In-Depth Study on Tribological Behavior Under Dynamic Sealing Conditions

Most existing studies focus on single-friction behavior in hydrogen environments, and there is still a lack of in-depth exploration of the fretting wear mechanism and its evolution law in high-pressure hydrogen environments. The high permeability and swelling property of hydrogen not only lead to the degradation of rubber’s mechanical properties, but may also induce microcrack propagation, interface failure, and early sealing damage. It is urgent to design a friction and wear test device for high-pressure hydrogen environments and conduct in-depth research on the fretting wear mechanism in high-pressure hydrogen environments.

#### 5.2.4. Impact of Damage Accumulation on Rubber Permeation Performance

Table 2 summarizes the key problems, research objectives, and expected outcomes for multi-mechanism, multi-physics failure modeling of sealing systems in high-pressure hydrogen, guiding long-duration testing and material/design optimization. In existing studies, the hydrogen permeation performance of rubber is generally regarded as an inherent property of the material, and it is assumed to remain stable throughout the service life. However, as seals operate for a long time in high-pressure hydrogen environment—especially when blister damage and microcrack propagation occur during multiple hydrogen charging–discharging cycles or rapid depressurization processes—the microstructure of O-rings will undergo continuous evolution, and this process exerts a profound impact on the permeation behavior. Combining mesoscopic micro-pore monitoring with machine learning can improve damage prediction accuracy.

## 6. Conclusions

This review examines the failure modes of rubber O-rings in high-pressure hydrogen environments and their influencing factors, outlining the transition from conventional high-pressure conditions to hydrogen-specific mechanisms. It highlights the distinctive features of newly emerged modes such as hydrogen-induced swelling and rapid gas decompression (RGD), as well as the amplification effects of traditional failure mechanisms. Furthermore, this paper identifies existing gaps in the literature, including insufficient studies on multi-mechanism coupling, a lack of long-term dynamic experimental data, and limitations in current life prediction models. By summarizing recent progress and proposing future research directions, this work provides a comprehensive theoretical foundation and practical engineering guidance for material development, structural optimization, and the formulation of standards for reliable sealing systems in hydrogen energy applications.

## Figures and Tables

**Table 1:** Performance requirements (operating pressure, cycle count, sealing effect, and leakage rate) of O-rings in high-pressure hydrogen environments.

Application Scenario	Working Pressure	Temperature Range	Requirements for Leak Rate and Cycle Count
On-board Hydrogen Storage Tank (Type IV)	70 MPa	−40~85 °C	Type A1, B1: No leakage for 15-year service life; Type A2, B2: No leakage during regular inspection period
Valve for Hydrogen Refueling Station	70 MPa	−40~85 °C	Leakage rate ≤ 10 cm^3^/h; Withstand 16,000 cycles
Hydrogen Gun Refueling Nozzle	98 MPa	−40~85 °C	Leakage rate ≤ 20 cm^3^/h; Withstand 16,000 cycles
High-pressure Hydrogen Storage Vessel	70 MPa	−40~85 °C	Service life: 100,000 cycles (15 years, with regular inspection)

**Table 2:** Research and development of multi-mechanism coupling failure models for sealing systems in high-pressure hydrogen environments.

Content	Explanation
Main Problems	Existing studies have four main limitations: overemphasis on single failure modes (lacking systematic research on multi-mechanism coupling), short-term experimental data (insufficient long-term support), numerical simulations ignoring multi-physics field coupling effects, and inadequate exploration of material design and temperature rise effects.
Research Objectives	Study multi-mechanism coupling failure patterns, conduct long-duration and multi-condition tests, develop multi-physics coupling models, create new sealing materials, and optimize design and operating conditions to enhance the reliability of sealing systems in high-pressure hydrogen environments.
Expected Outcomes	Systematically analyze the interrelationships between different failure modes, accumulate experimental data and failure evolution patterns in high-pressure hydrogen environment, establish a life prediction model for sealing systems in hydrogen energy equipment, and propose optimization design solutions based on experimental and simulation results.

## Data Availability

No new data were created or analyzed in this study. Data sharing is not applicable to this article.

## Abbreviations

The following abbreviations are used in this manuscript:

RGD	Rapid Gas Decompression
NBR	Nitrile Butadiene Rubber
HNBR	Hydrogenated Nitrile Butadiene Rubber
FKM	Fluoroelastomer
EPDM	Ethylene Propylene Diene Monomer
